# A Review of Melanoma Subtypes: Genetic and Treatment Considerations

**DOI:** 10.1002/jso.27953

**Published:** 2024-10-16

**Authors:** Christina Druskovich, Jesse Kelley, Jason Aubrey, Leah Palladino, G. Paul Wright

**Affiliations:** ^1^ College of Human Medicine Michigan State University Grand Rapids Michigan USA; ^2^ Department of General Surgery Corewell Health Grand Rapids Michigan USA; ^3^ College of Literature, Science, and the Arts University of Michigan Ann Arbor Michigan USA; ^4^ Department of Surgical Oncology Corewell Health Grand Rapids Michigan USA

**Keywords:** genetic markers, genetic mutations, melanoma genetic mutations, melanoma subtypes

## Abstract

Melanoma affects over one million people in the United States. This review explores genetic mutations and markers of all seven subtypes. Current treatment options and prognosis of each subtype are also discussed. For lentigo maligna, spitzoid, and nodular subtypes, BRAF was the most common mutation reported. For superficial spreading, TP53 was the most common. Acral lentiginous demonstrated CCDN1 and desmoplastic NF1 most frequently. No mutations have been identified in the nevoid subtype. Nodular melanoma is the deadliest subtype. Evidence suggests that the subtypes differ in regard to genetic markers/mutations, treatment and prognosis. Therefore, subtype should be considered when treating a melanoma patient.

## 1 Introduction

Melanoma affects over one million people in the United States and the incidence has been steadily increasing over the last decade [1]. Although melanoma is the third most common type of skin cancer, it accounts for the majority of skin cancer deaths, with 7990 deaths occurring in 2023 alone [2]. Risk factors include ultraviolet radiation exposure, fair skin, a history of sunburn, living close to the equator or at high altitudes, having more than 100 moles, family history of melanoma, personal history of dysplastic nevi, or a weakened immune system [3].

The time it takes for an in situ melanoma to metastasize varies, with some invading in just 1 month, but others taking greater than 5 years [4]. There is no single pattern in the development of melanoma; each subtype can involve different mutations and precursors [5]. The World Health Organization classified nine melanoma pathways in 2018 according to cumulative sun damage (CSD) as well as associated genetic mutations [6]. There are seven subtypes of melanoma, which include superficial spreading, nodular melanoma (NM), lentigo maligna, acral lentiginous, nevoid, spitzoid, and desmoplastic. Low‐CSD melanoma includes the subtype superficial spreading. High‐CSD melanoma includes the subtypes lentigo maligna and nodular. Non‐CSD melanoma features the acral subtype [7]. An understanding of melanoma subtypes can aid the surgeon in treatment decision making, patient counseling, and prognostic determination.

Treatment for each subtype begins the same, with wide local excision for most early‐stage lesions. For lesions with a Breslow depth less than 1 cm, margins of 1 cm are recommended. For lesions greater than 1 mm but less than 2 mm, margins of 1 to 2 cm are recommended. Margins of 2 cm are recommended in lesions greater than 2 mm [8] (Table 1). Staged excision and Mohs are other surgical techniques that may be options in select patients, but they continue to be the focus of studies on optimal technique for melanoma resection [9].

**Table 1:** Journal of the American Academy of Dermatology and American Joint Committee on Cancer guidelines for cutaneous melanoma margins.

Tumor thickness	Surgical margin
In situ	0.5–1 cm^a^
≤ 1.0 mm	1 cm
> 1.0–2.0 mm	1–2 cm
> 2.0 mm	2 cm

^a^ Excluding the LMM subtype.

The use of sentinel lymph node biopsy (SLNB) remains a hot topic. When deciding whether or not to perform a SLNB, a few different resources can be utilized to make a decision. The American Society of Clinical Oncology and the Society of Surgical Oncology publish guidelines as one entity that uses the traditional measures of Breslow depth and ulceration [10]. The National Comprehensive Cancer Network publishes recommendations based on Breslow depth, ulceration, high mitotic rate, and lymphovascular invasion [11]. Risk calculators are also available. The Memorial Sloan Kettering Cancer Center features a “melanoma nomogram” that calculates tumor spread to the sentinel lymph nodes. Variables in this calculation include age, tumor thickness, Clark level, melanoma location, and ulceration [12]. Similarly, the Melanoma Institute of Australia published their sentinel node metastasis risk calculator. Their calculations incorporate age, tumor thickness, melanoma subtype, mitoses, ulceration, and lymphovascular invasion [13]. Lastly, gene expression profiling tools are available, such as DecisionDx, but the National Comprehensive Cancer Network currently recommends against using this tool to make decisions on SLNB. In light of this, a multi‐institutional clinical trial is underway, named DecisionDx‐Melanoma Impact on SLNB Decisions and Clinical outcomes (DECIDE), which aims to evaluate outcomes of patients that used genomic stratification versus those who did not, in accordance with other standard variables [14].

Lymph node positivity tends to have worse prognosis than negative involvement. When comparing patients with intermediate thickness melanomas, 1.2–3.5 mm at 10 years, the melanoma‐specific survival rate was significantly worse in patients with node involvement compared to those with negative SLNB. Similarly, the melanoma‐specific survival rate at 10 years for those with thick melanoma, greater than 3.5 mm, was also significantly worse in patients with node involvement versus those with negative SLNB [15]. SLNB does not come without possible complications. Wound infection, lymphedema, hematoma/seroma, and sensory nerve injury are all possible complications. However, SLNB had much fewer of these complications when compared to regional lymphadenectomy [16].

In cases of advanced disease, immunotherapy (such as PD‐1 and anti‐CTLA‐4) and targeted therapy (i.e., BRAF inhibitors) can be utilized. Patients with resectable cutaneous melanoma can benefit from adjuvant or neoadjuvant therapy. Factors to consider when deciding to add such therapy include risk of disease recurrence, stage at diagnosis, BRAF mutation status, degree of lymph node involvement, treatment preferences, and comorbidities [17]. When applicable, patients can enroll in clinical trials. Stratification by subtype provides less value in the metastatic setting; however, a tendency toward certain molecular targets may have impact. Growing evidence for different genes and tumor markers provide possibly more treatment targets for each subtype. Herein we provide a review of pathologic subtypes of melanoma, focusing on the unique clinicopathologic characteristics and molecular features that can help to guide decision‐making and prognostic conversations in the care of the melanoma patient.

## 2 Superficial Spreading Melanoma (SSM)

The most common subtype of melanoma in Western countries is SSM [18], which accounts for approximately 70% of melanomas [19]. The current incidence of SSM is estimated to be six per 100 000 patients and is expected to increase in the coming years. Incidence is positively correlated with increasing age; patients between 70 and 79 years old have the highest incidence. Males had a higher incidence than females [20]. SSM is a low‐CSD melanoma characterized by slow horizontal growth. SSM are most common in people with fair skin, and present as flat and irregular lesions with black or brown coloration [21]. In females, SSM is more frequently detected on the lower limbs, whereas in males, it is most frequently found on the back [22]. In most cases (78.9%), Breslow thickness is less than 1 mm. Interestingly, low education, increased age, and rural living were found to be risk factors for increased Breslow depth at time of diagnosis, likely secondary to delays in diagnosis [23].

Several genetic mutations have been found within the SSM subtype. BRAFV600E is most frequently detected, with a majority also showing TERT promoter mutations and CDKN2A. One study found that 64.3% of SSM possessed BRAF mutations [24]. Less commonly, CCDN1 is found in about 6% of SSM [6] and PTEN and TP53 are observed in more advanced tumors [6]. TP53 was observed in 73% according to one study [25]. SSMs are often found on the trunk and proximal areas of the extremities and diagnosed as thinner lesions [6, 26].

If advanced or metastatic disease is present, adjuvant therapy targeting specific mutations could be considered, particularly, BRAFV600E/K inhibitors, MEK1/2 inhibitors, or a combination therapy. However, this should be left to the discretion of the provider, as these decisions are multifactorial. The BRAFV600E mutation is frequently tied with SSM. A combination of dabrafenib plus trametinib is approved for stage III BRAFV600E/K melanoma [27]. Other FDA approved combinations for BRAF V600 include vemurafenib/cobimetinib and encorafenib/binimetinib [27]. The prognosis for SSM is quite good, with a 5‐year survival of 95% [20]. Patients with tumors equal to or less than 1 mm thick, local lesions, and no ulceration had higher survival than those with opposing features [20].

## 3 Nodular Melanoma (NM)

NM is the second most common type of melanoma, comprising 14%–15% of all cutaneous melanoma cases [28]. The incidence of death in NM is higher in male sex patients and have tumors classified as a high‐risk thickness, specifically greater than 2 mm [29]. NMs often feature dark pigmentation, pedunculated or polypoid nodules. The clinician should be aware of amelanotic variants, as they can lead to delays in diagnosis; however, they are rare [30].

Unlike SSM, NM commonly develops in sun‐exposed areas and takes only weeks to months to develop [29]. In men, NM is most commonly found on the head, neck, and back area, whereas for women, NM is most commonly found on the legs [31]. Oftentimes it presents as a smooth, hyperpigmented, well‐circumscribed, raised lesion, but can also be hypomelanotic or amelanotic [29]. Patients often endorse bleeding, itching, or stinging at the site [29]. Clinically, its presentation can resemble that of basal cell carcinoma, viral wart, seborrheic keratosis, pyogenic granuloma, or even a fibroma [29].

The BRAF mutation is also associated with NM, with an estimated prevalence of 36.4%–47% [24, 32]. One study demonstrated that strong BRAF‐V600E protein expression was associated with aggressive tumor features, such as increased thickness and presence of ulceration [33], likely contributing to its reduced survival. However, NM is more frequently associated with upregulated PD‐L1, the NRAS mutation and decreased tumor‐infiltrating lymphocytes, but the exact pathogenesis of its aggressive behavior is unknown [34]. NRAS was found to have a 27% mutation rate in one study [35]. Compared to other subtypes, NM was 6.48 times more likely to be positive for PD‐L1 [36].

Growing evidence demonstrates that NM is the deadliest subtype of melanomas, responsible for about 40% of melanoma deaths [28]. The aggressive nature of NM can be contributed to the lack of prodromal radial growth phase, faster growth rate, greater mitosis, greater Breslow thickness compared to other subtypes [28], absence of regression [29] and early distant metastasis. The rapid, malignant growth of this subtype can make it fatal within months of recognition [31]. When comparing SSM and NM, no significant difference between the two was found in overall survival after starting immune‐checkpoint inhibition therapy [34]. However, when corrected for Breslow thickness and ulceration, NM had a significantly decreased 5‐year relative survival compared to SSM, at 61.5% versus 89.7% [26]. This difference in relative survival could be explained by the shorter distance‐free metastasis interval of NM. It is speculated that the subtype was the underlying cause of this difference in survival, likely guided by tumor genetics and characteristics. Thus, subtype should be considered in the overall assessment of patients when deciding to add adjuvant therapy [34].

## 4 Lentigo Maligna Melanoma (LMM)

Lentigo maligna is a melanoma in situ, often mistaken for benign lesions. About 5%–20% of these will progress to an invasive melanoma, termed LMM [37, 38]. For the purposes of this paper, we will focus on the LMM subtype. LMM makes up 4%–15% of all melanomas, making it the third most common subtype [39]. In addition to being a high‐CSD melanoma [40], risk factors include fair skin, personal history of skin cancer, and the quantity of solar lentigines and keratoses [41].

LMM is oftentimes located on the head and neck, representing 78.3% of cases [42]. LM and LMM are typically a large, isolated, and slowly growing pigmented macule or patch with irregular, ill‐defined borders. These lesions can be amelonotic or hypomelanotic [43]. Mutations associated with LMM include CCDN1, which is found in about 11% of LMMs [44]. NRAS, TERT, KIT, BRAFnonV600E, PTEN, CDKN2A, and TP53 are also associated mutations [6]. BRAF was found to have a 53.4% mutation rate [24]. In one study, TP53 was mutated in 52% of LMMs [25].

It is important to note that for LMM, margins of 0.5 cm are not acceptable [8, 45]. Besides this exception, the LMM subtype follows the same guidelines published by the American Academy of Dermatology (AAD) as other cutaneous melanoma types. For cosmetically sensitive areas and LMM less than 0.8 mm Breslow thickness, Mohs surgery is a viable option [46]. Stage 1A LMM can be managed with staged excision, while stage 1B and beyond are referred for wild local excision [47]. However, depending on patient demographics, preference, compliance, and lesion characteristics, additional therapies can be considered, such as radiotherapy, cryosurgery, laser therapy, and topical therapy [48]. Although studies have not verified imiquimod use for LMM as standard of care option, it may be considered in poor surgical candidates [47]. Surgical management is the preferred approach as nonsurgical methods have been demonstrated to have a higher rate of recurrence [49]. An exception would be elderly patients with multiple comorbidities or where resection could cause significant deformity. The 5‐year prognosis for local LMM is 100%, for LMM that spread to sentinel lymph nodes or nearby tissues, it is 73.2%, and for distant metastasis, it is 26.1% [10].

## 5 Acral Lentiginous Melanoma (ALM)

ALM is a form of melanoma that occurs on the hand and feet, specifically, the palms, soles, fingers, toes, and nails [50]. ALM can present as a pigmented macule characterized by an irregular border with varied pigment. Lesions can be mistaken for wounds, warts, hematomas, or other benign lesions. On the nail, ALM can present as diffuse pigmentation, a pigmented stripe, or other changes not containing pigment, such as ulceration [51]. It is distinguished by a radial growth phase that evolves to an invasive, vertical growth stage [52]. Compared to other subtypes of melanoma, ALM is one of the less common types and consists of only 3% of all melanoma diagnoses though the incidence has been increasing steadily [53]. However, unlike other melanoma subtypes, ALM is often more advanced at the time of presentation likely owing to the difficulty in diagnosis, boasting a greater morbidity and mortality. It also has the highest rate of nodal metastasis among the subtypes. The development of ALM is not believed to be affected by ultraviolet radiation [50].

Mutations affecting ALM include CCDN1 gene amplifications in about 30% of cases and CDK4 is also frequently found. The C‐KIT mutation is found in ALM in about 10%‐15% [44]. NRAS was found in 15% of ALM and PTEN was identified in 4% [54]. NTRK translocations have been identified in ALM [55]. NRAS, BRAF, ALK rearrangement, CDKN2A and TERT have also been noted in ALM [6]. TERT had a frequency of 6% [56]. Notably, BRAF and NRAS are found much less frequently in ALM than other cutaneous melanomas [44]. BRAF demonstrated a mutation rate of 9.5% in one study [24]. In a deep‐sequencing study, PRKCA was identified in 0.8%, NF1 in 14.8%, PAK1 and GAB2 in 22.1%, CDK4 in 22.1%, EP300 in 16.4%, YAP1 in 12.3%, MDM2 in 10.7%, and TERT in 10.7% [55].

Large excisions on acral surfaces can be hard to close and can involve digital amputations, warranting the use of grafting when necessary [50]. BRAF and MEK in combination have proven to work well in BRAF‐mutated melanomas, but because of the low frequency of mutation among ALM, it has limited efficacy in this subtype [44]. It is estimated that the frequency of the BRAF or NRAS mutations in ALM are 10%‐15% [57]. Inhibitors against PI3K/Akt/mTOR, CDK, and MDM2/p53 are currently being explored as potential treatment options [58]. ALM patients, when compared to other forms of melanoma patients, demonstrated significantly shorter overall survival with CTLA‐4, PD‐1, or PD‐L1 inhibitors [59]. Radiation treatment can be used for adjuvant treatment, palliation, and for patients with recurrent disease [60]. For systemic therapy treatment, imiquimod, darcabazine, and interferon may increase the ability of the immune checkpoint inhibitors to function by increasing tumor‐infiltrating lymphocytes [61].

Compared to other subtypes of melanoma, ALM disproportionately affects nonwhite populations with Black patients having the worst prognosis [53, 62]. The 5‐year survival rate is 80.3%, and the 10‐year survival rate is 67.5%, less than its cutaneous melanoma counterparts [63].

## 6 Desmoplastic Melanoma (DM)

Accounting for less than 4% of melanomas, DM is one of the less frequent subytpes [64]. With a strong association with chronic sun exposure, it is often found later in life, between the ages of 60–80 years old and has a predilection for the head and neck [65]. DM affects females to males with a 2 to 1 ratio. DM presents as amelanocytic nodules or plaques, and can resemble other benign dermatological findings, including scars, dermatofibromas and neurofibromas. DM also may resemble malignant lesions, such as basal cell carcinoma, squamous cell carcinoma, and amelanocytic melanoma [66]. DM is a variant of spindle cell melanoma and can be mistaken for other benign and malignant spindle cell tumors, further making it a difficult diagnosis [66]. It is divided into 2 subgroups: pure and mixed. The pure subtype encompasses a pure invasive component (>90%), while the mixed has features of DM and non‐DMs, leads to worse survival outcomes, and has more frequent metastasis [66].

In 75% of DM cases, the TP53 gene was found to be mutated [67]. NF1 was mutated at a high rate, at 93% [68]. Less frequently, neurofibromin 2, cyclin‐dependent kinase inhibitor 2A and AT‐rich interactive domain‐containing protein 2 were notable mutations [69]. NF2 specifically was found in 2/12 cases [69]. Further, mixed DM associated with SSM possessed a BRAF V600E mutation and an acral DM reported with a c‐kit mutation. Hayward et al. noted NFKBIE promoter mutations, deletion of TCS1/TCS2, and activating mutations of NOTCH1 and KDR [70]. In a separate study, direct sequencing revealed RET in 33% of pure DM and 24% of mixed. Immunohistochemical staining identified numerous markers. Ki‐67 was 5% in pure and 28% in mixed, CD117 stained 26% in pure and 78% in mixed, nestin 83% in pure and 89% in mixed, clusterin 4% in pure and 6% in mixed, SOX10 87% in pure and 94% in mixed, and lastly, CD271 stained 61% in pure and 67% in mixed [71].

Treatment typically begins with surgical management, namely wide local excision. The AAD and American Joint Committee on Cancer guidelines are recommended (Table 1). A large case series study stated that margins should be at a minimum of 1 cm, because patients with 1‐cm margins or greater had a better overall survival than patients with margins less than 1 cm [72]. DM has an inclination for local recurrence, ranging from 6.7% to 56% [73].

SLNB has come into question for DM patients because of the low risk of sentinel node involvement and unclear survival benefits [72, 74]. The mixed DM subtype was found to have a higher nodal involvement than the pure subtype, at 18.5% and 1.4%, respectively [74]. Interestingly, a separate study found no affected nodes in DM patients [75]. Alternatively, it has been suggested to consider high‐risk factors, such as high mitotic rate, ulceration, and neurotropism, when deciding whether or not to perform SLNB [76, 77].

Historically, advanced DM is difficult to treat. Currently, a PD‐L1 inhibitor, pembrolizumab, has demonstrated to be useful for neoadjuvant use in DM [78]. A previous study by Eroglu et al. in 2018 [79] also demonstrated promising PD‐L1 inhibitor response, with 70% of patients having objective tumor response and 32% showing a complete response. Further analysis of mutations could provide future insight with targeted therapies.

Due to predominantly local recurrence, radiation has been utilized more frequently in the adjuvant setting in DM while other subtypes are generally considered to be radioresistant. One study observed that DM patients who underwent surgical resection with adjuvant radiation had greatly reduced recurrence rates compared to those who had surgical resection alone, at 0% and 57%, respectively [80]. The 5‐year overall survival for DM ranges between 67%–89% [72].

## 7 Nevoid Melanoma

Nevoid melanoma is rare, with an incidence estimated to be no more than 0.5% of melanomas [81]. Nevoid melanoma is proclaimed to be one of the most difficult melanomas to diagnose, as it is often mistaken for benign dermal nevi because of their similar appearance [82]. They can be verrucous or dome‐shaped nodules, typically found on the trunk or proximal limbs in young adults [83]. They may resemble papules or have plaque‐type topology. In addition to their resemblance of benign nevi, they lack the “ABCDEs” of melanoma, including asymmetry, irregular border, color, diameter, and evolution. Nevoid melanoma is further divided into 3 groups based on dermatoscopic features, nevus‐like, amelanotic, and tumors with multicomponent pattern. Tumors demonstrating a mixed pattern are not part of these classification groups [82]. The mean Breslow thickness for nevoid melanoma was 3.2 mm at the time of biopsy [84]. Breslow thickness is the most important predictor of prognosis in this subtype, but ulceration is also important to assess [85]. Currently, to the best of our knowledge, no data exists on genetic mutation frequency for nevoid melanomas. The AJCC staging prognostic estimates apply to this subtype, with stage I having a promising prognosis with 95% 5‐year overall survival, and stage IV demonstrating poor prognosis with a median survival of 6–9 months [85].

## 8 Spitzoid Melanoma

A rare and challenging diagnosis, the spitzoid melanoma typically emerges during childhood or young adulthood, but they can exist at any age [86, 87]. A recent Finish study discovered a four‐fold increase in melanoma incidence, with the spitzoid subtype having the most pronounced increase [88]. As the name implies, they mimic the benign Spitz nevus typically seen in children, both clinically and histopathologically. They tend to not follow the “ABCDE” rules of melanoma, further adding to diagnostic difficulty. The lateral diameter is often greater than 6 mm, and lesion size frequently surpasses 1 cm. Presentation includes nodules, papules and plaques; pigmentation is varied, with some studies claiming they are more often amelanocytic [86]. The lower extremities are the most common location of the neoplasm [89]. Interestingly, Spitz nevi often have benign deposits in regional nodes that do not impact prognosis as metastatic regional nodes do in melanoma. Spitzoid melanoma maintains similar nodal metastasis rates to other subtypes of melanoma.

BRAF and NRAS mutations were found in 20% of spitzoid melanomas in one study, which was lower than the 61% in nonspitzoid melanoma [90]. Other studies also confirm a lower rate of BRAF and NRAS mutations in spitzoid melanoma compared to other melanoma subtypes [91]. HRAS mutation was not found to be in spitzoid melanomas [92]. Translocations involving the kinases ALK, ROS1, MET, RET, BRAF, and NTRK1 were observed in 50% of spitzoid melanoma cases, but not common in other types [68].

While management of Spitz nevi has been a topic of controversy, spitzoid melanoma follows the guidelines of other melanoma, treatment depends on Breslow thickness and Clark's level. Treatment begins with wide local excision and follows AAD and AJCC guidelines (Table 1). SLNB follows the same guidelines as for other melanoma subtypes. SLNB is recommended for tumors 1 mm or greater in Breslow thickness. In regard to other therapy adjuvants, high dose interferon therapy followed by maintenance therapy demonstrated prolonged recurrence‐free survival by 37%; however, this has fallen out of favor in the era of checkpoint inhibitor therapy [91]. For advanced disease, immunotherapy, targeted therapy, radiation, or a combination of therapies can be used [93].

Compared to other melanoma subtypes with the same Breslow thickness, spitzoid melanoma carries the same prognosis. Other prognostic factors include thickness of primary tumor, presence of ulceration, and nodal status. Age may be a positive prognostic factor in childhood spitzoid melanomas. For children ages 0–10 years old, the 5‐year survival rate was 88%, compared to 49% in children 11–17 years old [91].

## 9 Conclusion

This review of published literature outlines genetic mutations and markers identified in each subtype of melanoma, as well as prognosis, and key points in current treatment options. This review bridges a gap with concise findings regarding up‐to‐date information involving genetics in melanoma subtypes. Differences in the genetic background and disease course are highlighted in this paper. Future directions include further research on genetic markers or mutations, and treatments or treatment targets for the melanoma subtypes (Tables 2 and 3).

**Table 2:** A summary of genetic mutations or markers and the rate at which they were found.

Subtype	Gene/marker	Mutation rate
Superficial spreading	TP53	73%
	BRAFV600E, TERT, CDK2A	64.3%
	CCDN1	6%
	PTEN	Unspecified
Nodular	BRAF	36.4%–47%
	NRAS	27%
	PD‐L1	Unspecified
Lentigo maligna melanoma	BRAF	53.4%
	TP53	52%
	CCDN1	11%
	NRAS, TERT, KIT, BRAFnonV600E, PTEN, CDKN2A	Unspecified
Acral Lentiginous	CCDN1	30%
	TERT	10.7%–44.9%
	CDK4	22.1%
	PAK1, GAB2	22.1%
	EP300	16.4%
	NRAS	15%
	C‐KIT	10%–15%
	YAP1	12.3%
	MDM2	10.7%
	BRAF	9.5%
	TERT	6%
	PTEN	4%
	NTRK3 translocation	2.5%
	PRKCA	0.8%
	ALK, CDKN2A	Unspecified
Desmoplastic	NF1	93%
	SOX10	87% (pure), 94%(mixed)
	Nestin	83%(pure), 89%(mixed)
	TP53	75%
	CD271	61%(pure), 67%(mixed)
	TERT	34%
	RET	33% (pure subtype), 24% (mixed subtype)
	CD117	26% (pure subtype), 78% (mixed)
	NF2	16.6%
	Ki‐67	5%(pure), 28% (mixed)
	Clusterin	4%(pure). 6%(mixed)
	NF2, cyclin‐dependent kinase inhibitor 2A, AT‐rich interactive domain‐containing protein 2, c‐kit NFKBIE, TCS1/TCS2, NOTCH1, KDR	Unspecified
Nevoid	None known	Not applicable
Spitzoid	BRAF, NRAS	20%
	Translocations: ALK, ROS1, MET, RET, BRAF, NTRK1	50%

**Table 3:** Key points for the clinician of each melanoma subtype.

Subtype	Key points
Superficial spreading	BRAFV600E, TERT, and CDKN2A are the most common mutations Most common subtype
Nodular	Deadliest subtype, accounting for 40% of melanoma deaths Frequently associated with BRAF, PD‐L1, and NRAS mutations
Lentigo maligna	High‐CSD melanoma, majority present on the head and neck CCDN1 is the most common mutation 0.5‐cm margins are not acceptable for this subtype
Acral lentiginous	Disproportionately affects Black patients, often a worse prognosis CCDN1 and CDK4 are most frequently found
Desmoplastic	TP53 is most frequently mutated Relatively high recurrence rate
Nevoid	Difficult to diagnose because it can resemble dermal nevi No known genetic mutation associations
Spitzoid	BRAF and NRAS mutations are frequently identified Children aged 11–17 have a low 5‐year survival rate

## Author Contributions

**G. Paul Wright:** conceptualization, writing–review and editing, supervision. **Christina Druskovich:** writing– original draft, writing–review and editing. **Jesse Kelley:** writing–review and editing, supervision. **Jason Aubrey:** writing–review and editing, supervision. **Leah Palladino:** writing–review and editing.

## Synopsis

This review provides a comprehensive summary of histologic melanoma subtypes, covering genetic mutations, prognosis, treatment, and notable considerations for each.

## Data Availability

Data sharing not applicable to this article as no datasets were generated or analyzed during the current study.
